# Impact of Patent Foramen Ovale on Ischemic Stroke Outcomes in Patients Aged 65 and Older: A Retrospective Cohort Study

**DOI:** 10.7759/cureus.98155

**Published:** 2025-11-30

**Authors:** Nouraldeen Manasrah, Abdelrahman Zaied, Caroline Shaver, Samah Ahmadieh, Rohan Sharma, Samarth Mishra, Sarah Alqasem, Wael Aljaroudi

**Affiliations:** 1 Cardiology, Augusta University Medical College of Georgia, Augusta, USA; 2 Internal Medicine, Hospital Corporation of America (HCA) Florida JFK Hospital, Atlantis, USA; 3 Internal Medicine, Augusta University Medical College of Georgia, Augusta, USA; 4 Hematology, Augusta University Medical College of Georgia, Augusta, USA; 5 Internal Medicine, Luzmila Hospital, Amman, JOR

**Keywords:** elderly patients, ischemic stroke, patent foramen ovale (pfo), readmission rate, rope score

## Abstract

Introduction

This study evaluated the clinical characteristics, outcomes, and management implications associated with patent foramen ovale (PFO) in patients aged 65 and older who presented with ischemic stroke at a single center.

Methods

This retrospective study included patients aged 65 and older who were admitted with ischemic stroke between 2017 and 2019 and were followed for three years. Patients were categorized based on the presence or absence of a PFO identified during the index hospitalization. Outcomes included events during the index admission and ischemic stroke readmissions during follow-up. Continuous variables were analyzed using the Student’s t-test, and categorical variables were compared using the Pearson Chi-square test. Binary logistic regression was used to identify independent predictors of ischemic stroke recurrence.

Results

A total of 1,090 patients were included. Of these, 116 patients (10.6%) had a PFO and 974 (89.4%) did not. The mean age was 74.8 years in the PFO group and 75.9 years in the non-PFO group. Comorbidities were similar between groups. The three-year ischemic stroke readmission rate was 8.9% in the PFO group and 11.2% in the non-PFO group (P = 0.36). Hypertension was independently associated with ischemic stroke recurrence (Odds Ratio = 3.88; 95% CI: 1.1-17; P = 0.04). No patients with PFO underwent closure during follow-up.

Conclusion

Among patients aged 65 and older admitted with ischemic stroke, the presence of PFO was not associated with differences in three-year stroke readmission rates or length of stay during the index hospitalization. In this cohort, ischemic stroke recurrence was associated with hypertension rather than PFO status.

## Introduction

Ischemic stroke remains a leading cause of morbidity and mortality among individuals aged 65 and older. In the United States, ischemic strokes account for approximately 87% of all strokes each year, with nearly 75% occurring in individuals over the age of 65 [1]. This population bears a disproportionate burden of stroke-related disability and death, making ischemic stroke a critical public health concern. The increasing longevity of global populations has contributed to a rise in both the incidence and prevalence of ischemic stroke among older adults, reinforcing the need for age-specific diagnostic and therapeutic frameworks.

The foramen ovale is a fetal cardiac structure that facilitates oxygenated blood flow across the atrial septum in utero. In approximately 75% of individuals, this communication spontaneously closes during the neonatal period. However, in an estimated 15-35% of the population, the foramen ovale remains patent, resulting in a condition known as patent foramen ovale (PFO) [2]. While the prevalence of PFO decreases with age, the average anatomical size of persistent PFOs appears to increase [3]. PFO has been identified in up to 50% of patients with cryptogenic stroke, highlighting its clinical relevance in cases of stroke with no clear etiology, including embolic stroke of undetermined source [4]. Diagnosis of PFO is based on the detection of a right-to-left shunt, achieved through contrast-enhanced transthoracic echocardiography (TTE), transesophageal echocardiography (TEE), or transcranial Doppler (TCD) using agitated saline [4].

The role of PFO in younger patients (<65 years) with cryptogenic stroke has been well studied [5]. However, its clinical significance in older adults remains unclear, particularly given the increased prevalence of coexisting stroke risk factors in this population. The presence of a PFO does not establish a causal relationship with stroke and may represent an incidental finding. The Risk of Paradoxical Embolism (RoPE) score is a validated tool used to estimate the likelihood that a stroke is attributable to a PFO; higher scores suggest a stronger association [4]. In younger patients with high-risk PFO anatomy, device closure has been shown to reduce the risk of recurrent stroke [5]. However, the benefits of PFO closure in patients over the age of 60 are less established. As a result, the clinical impact of PFO in older stroke patients remains poorly defined.

This study aims to evaluate the clinical characteristics, outcomes, and management implications of PFO in patients aged 65 and older presenting with ischemic stroke.

## Materials and methods

Study design

This is a single-center, retrospective study that included patients aged 65 and older who were admitted with ischemic stroke between 2017 and 2019 and were followed for three years. The study was approved by the Institutional Review Board (IRB).

Study population

Patients were identified using ICD-10 codes [6] for ischemic strokes and Patent Foramen Ovale. Patients were excluded from the analysis if they were less than 65 years old. Subsequently, patients were stratified into two cohorts based on the presence of PFO.

Patient characteristics

Baseline patient characteristics, including demographics and clinically relevant comorbidities, are shown in Table 1. Our study included all patients admitted for ischemic stroke with and without PFO, aged >65 years. Relevant comorbid conditions (i.e., diabetes, hypertension, hyperlipidemia, Atrial fibrillation, smoking, CKD, ROPE score) were collected.

**Table 1:** Baseline characteristics stratified by the presence of PFO and ROPE score RoPE: Risk of Paradoxical Embolism

	All Patients (N=1090)	PFO present (N=116)	PFO absent (N=974)	P
Demographics				
Age, mean (SD), yrs	75.9 (8.1)	74.8 (7.2)	75.9 (8.2)	0.12
Age, categorical				0.88
65-75 yrs	364 (33.4%)	38 (32.8%)	326 (33.5%)	
>75 yrs	726 (66.6%)	78 (67.2%)	648 (66.5%)	
Female	555 (50.9%)	61 (52.6%)	494 (50.7%)	0.70
Caucasian	621 (57%)	60 (51.7%)	561 (57.6%)	0.23
Comorbidities				
Hypertension	1013 (92.9%)	107 (92.2%)	906 (93%)	0.76
Diabetes	500 (45.9%)	54 (46.6%)	446 (45.8%)	0.88
Atrial fibrillation	364 (33.4%)	32 (27.6%)	332 (34.1%)	0.16
Dyslipidemia	556 (51%)	61 (52.6%)	495 (50.8%)	0.72
Chronic kidney disease	198 (18.2%)	22 (19%)	176 (18.1%)	0.81
Tobacco use	205 (18.8%)	27 (23.3%)	178 (18.3%)	0.19
ROPE score	3.49 (0.95)	3.40 (0.98)	3.5 (0.95)	0.28
ROPE score, categorical				0.89
0-3	502 (48.7%)	59 (50.9%)	443 (48.4%)	
4	416 (40.3%)	46 (39.7%)	370 (40.4%)	
5	97 (9.4%)	10 (8.6%)	87 (9.5%)	
6-8	16 (1.6%)	1 (0.9%)	15 (1.6%)	

Outcome measures

The primary outcomes were the three-year readmission rate and recurrent ischemic stroke among patients with and without PFO.

Statistical analysis

Continuous variables were expressed as mean ± standard deviation and compared using the two-tailed Student’s t-test for normally distributed data or the Wilcoxon rank-sum test for skewed data. Categorical variables were presented as frequencies and percentages and compared using the Pearson chi-square test. Binary logistic regression was performed to identify independent predictors of recurrent stroke at 3 years. To avoid model overfitting, variables with P < 0.2 on univariate analysis - including race, hypertension, dyslipidemia, and smoking - were included, and age and PFO were forced into the model.

Receiver operating characteristic (ROC) curve analysis was performed to assess the relationship between PFO and recurrent stroke. For sensitivity analysis, a Forest plot was constructed to display the adjusted odds ratio of PFO as a predictor of recurrent stroke across different patient subgroups. All statistical tests were two-tailed, with P < 0.05 considered statistically significant. Analyses were conducted using SPSS Statistics version 27 (IBM Corp., Armonk, NY, USA).

## Results

Patient characteristics

A total of 1,090 patients aged 65 and older who were admitted with ischemic stroke between 2017 and 2019 were followed for three years to assess the risk of recurrent stroke. Among them, 116 patients (10.6%) had PFO, while 974 (89.4%) did not. The mean age was 74.8 years in the PFO group and 75.9 years in the non-PFO group, with no significant differences in comorbidities between the two groups, as shown in Table 1.

Outcomes

The three-year ischemic stroke readmission rate was 8.9% among patients with PFO and 11.2% in those without PFO (P = 0.36). Hypertension was significantly associated with recurrent stroke (Odds Ratio = 3.88, 95% CI: 1.1-17; P = 0.04) as shown in Table 2. Notably, none of the patients with PFO underwent closure during the follow-up period.

**Table 2:** Baseline characteristics stratified by the recurrence of stroke at 3 years RoPE: Risk of Paradoxical Embolism

	All patients (N=1090)	Recurrent stroke within 3 years (N=97)	No recurrence of stroke within 3 years (N=993)	P
Demographics				
Age, mean (sd), yrs	75.9 (8.1)	74.1 (7.4)	76 (8.1)	0.025
Age, categorical				0.30
65-75 yrs	364 (33.4%)	37 (38.1%)	327 (32.9%)	
>75 yrs	726 (66.6%)	60 (61.9%)	666 (67.1%)	
Female	555 (50.9%)	49 (50.5%)	506 (51%)	0.93
Caucasian	621 (57%)	47 (48.5%)	574 (57.8%)	0.076
Comorbidities				
Hypertension	1013 (92.9%)	95 (97.9%)	918 (92.4%)	0.044
Diabetes	500 (45.9%)	41 (42.3%)	459 (46.2%)	0.46
Atrial fibrillation	364 (33.4%)	31 (32%)	333 (33.5%)	0.75
Dyslipidemia	556 (51%)	43 (44.3%)	513 (51.7%)	0.17
Chronic kidney disease	198 (18.2%)	15 (15.5%)	183 (18.4%)	0.47
Tobacco use	205 (18.8%)	25 (25.8%)	180 (87.8%)	0.066
Patent foramen ovale	116 (10.6%)	13 (13.4%)	103 (10.4%)	0.36
ROPE score	3.49 (0.95)	3.48 (0.83)	3.49 (0.97)	0.92
ROPE score, categorical				0.89
0-3	502 (48.7%)	46 (52.3%)	456 (48.4%)	
4	416 (40.3%)	34 (38.6%)	382 (40.5%)	
5	97 (9.4%)	7 (8.0%)	90 (9.5%)	
6-8	16 (1.6%)	1 (1.1%)	15 (1.6%)	
Readmission for stroke	97 (8.9%)	13 (11.2%)	84 (8.6%)	0.36

Predictors of recurrent stroke

**Figure 1:**
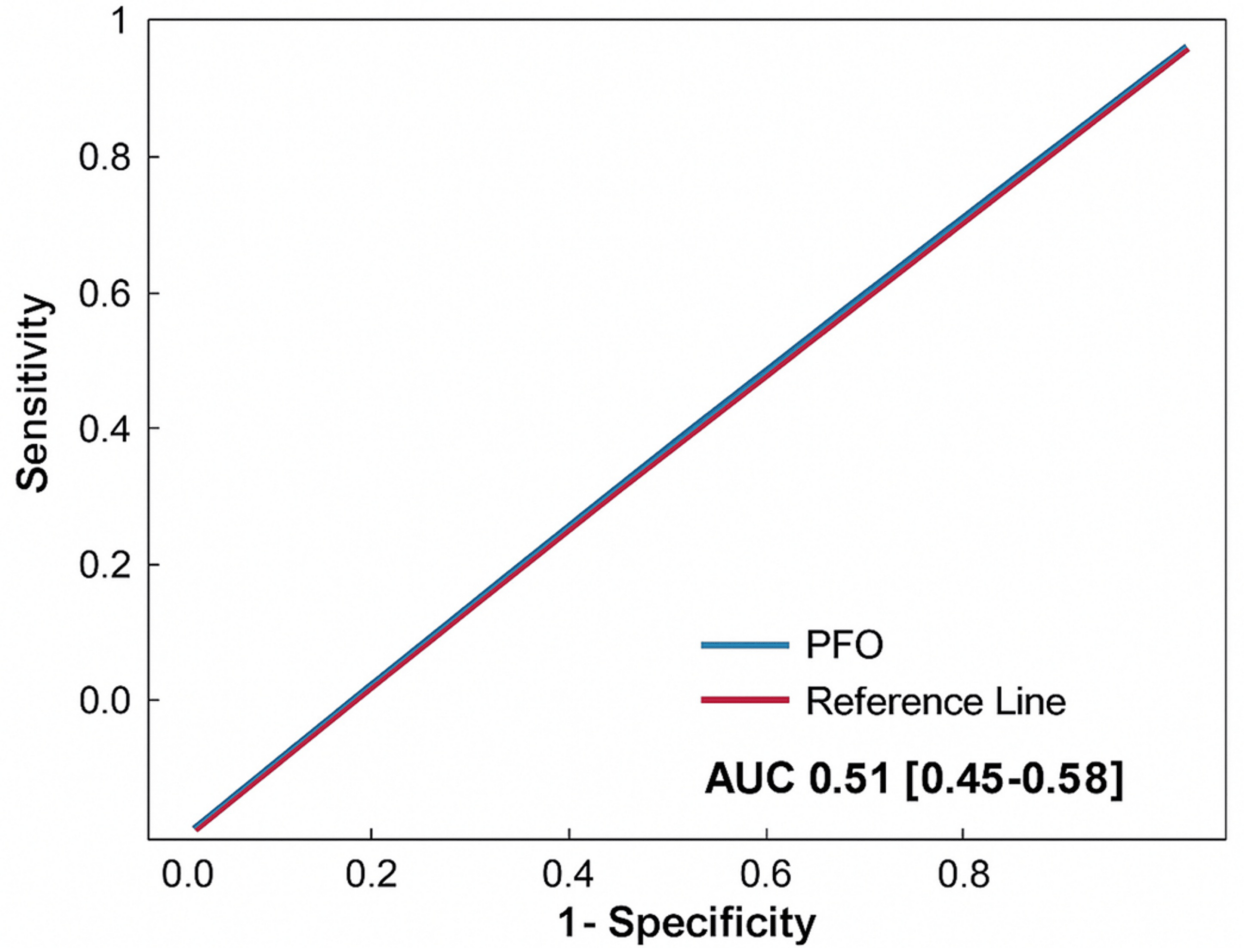
Receiving operator curve: PFO as predictor of recurrent stroke PFO: Patent Foramen Ovale

To evaluate whether PFO was associated with recurrent ischemic stroke at 3 years, we performed two complementary analyses. First, a receiver operating characteristic (ROC) curve was generated to assess the discriminative ability of PFO for predicting recurrent stroke (Figure 1).

**Figure 2:**
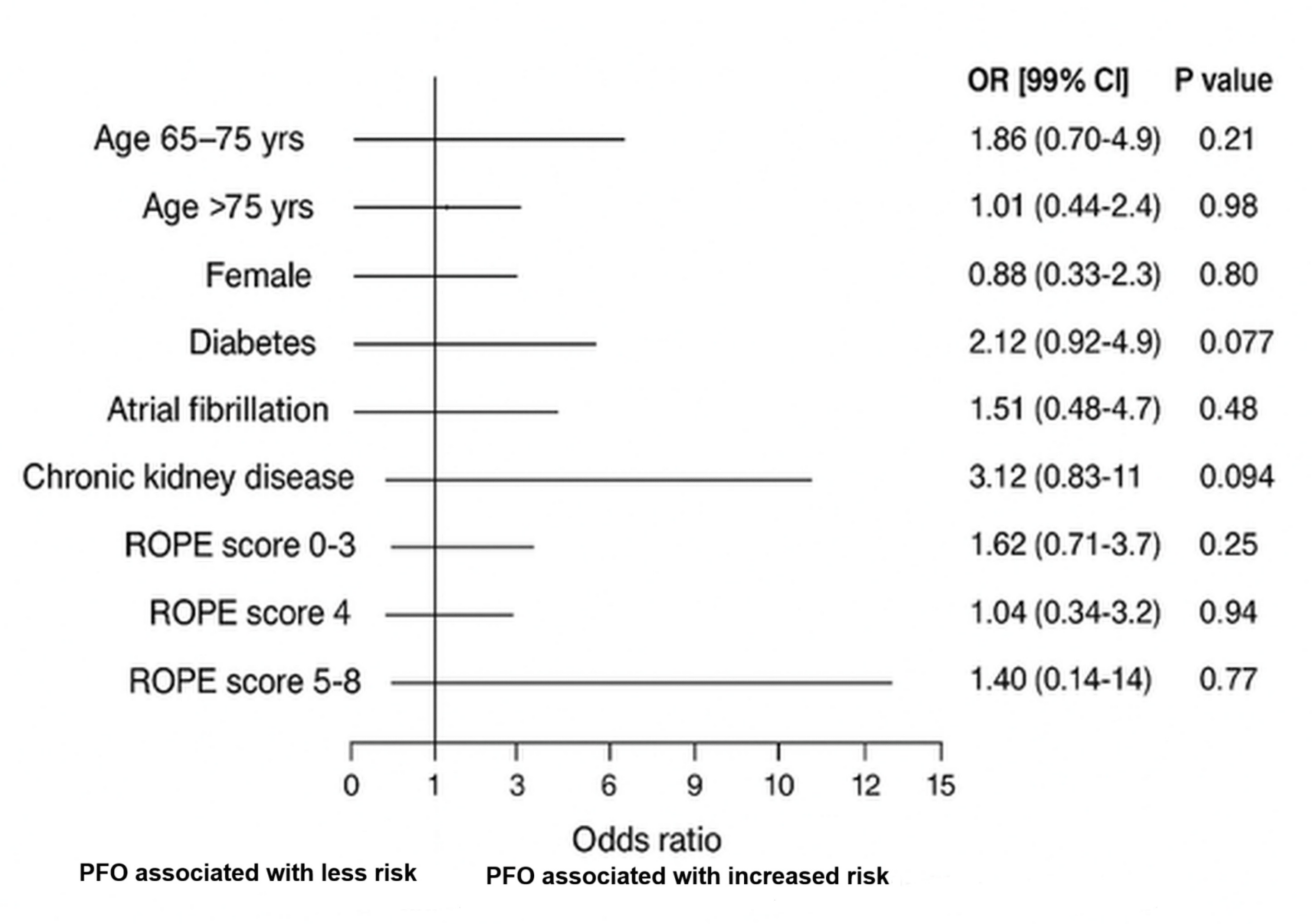
Adjusted odds of PFO as predictor of recurrent stroke RoPE: Risk of Paradoxical Embolism

Second, a multivariable logistic regression analysis was conducted, adjusting for significant univariate predictors (age, race, hypertension, dyslipidemia, and smoking), to estimate the odds of recurrent stroke associated with PFO across different patient subgroups (Figure 2, Table 3). These analyses allowed us to both visualize the predictive performance of PFO and explore potential subgroup effects.

**Table 3:** Predictors of recurrent stroke RoPE: Risk of Paradoxical Embolism

	Unadjusted			Adjusted		
	Odds ratio	95% CI	P	Odds ratio	95% CI	P
Demographics						
Age, mean (sd), yrs	0.969	0.942-0.996	0.026	0.975	0.946-1.004	0.088
Age >75 yrs	0.796	0.518-1.22	0.30			
Female	0.983	0.647-1.49	0.93			
Caucasian	0.686	0.452-1.04	0.077	0.81	0.52-1.23	0.31
Comorbidities						
Hypertension	3.88	0.938-16.05	0.061	4.24	1.01-17.8	0.048
Diabetes	0.852	0.559-1.29	0.46			
Atrial fibrillation	0.931	0.598-1.46	0.75			
Dyslipidemia	0.745	0.49-1.13	0.17	0.662	0.43-1.01	0.058
Chronic kidney disease	0.81	0.456-1/44	0.47			
Tobacco use	1.57	0.968-2.54	0.068	1.35	0.81-2.24	0.25
Patent foramen ovale	1.34	0.720-2.48	0.36	1.29	0.692-2.42	0.42
ROPE score	0.989	0.786-1.24	0.92			
ROPE score, categorical			0.89			
0-3	Ref					
4	0.882	0.555-1.40				
5	0.771	0.337-1.76				
6-8	0.661	0.085-5.12				

## Discussion

In our single-center retrospective analysis of 1,090 patients aged 65 and older admitted with ischemic stroke between 2017 and 2019, 116 individuals (10.6%) presented with PFO. Over a three-year follow-up period, the ischemic stroke readmission rate was not statistically different at 8.9% in the PFO group compared to 11.2% without PFO (P = 0.36). Hypertension emerged as the only significant predictor of recurrent stroke (OR = 3.88; 95% CI 1.1-17; P = 0.04). Notably, none of the PFO patients underwent closure during the study period.

Our study findings align with broader data showing that age and uncontrolled vascular risk factors are major contributors to stroke recurrence [7]. The RoPE score, which was developed primarily for younger patients, may not perform well in older adults. As age increases, the score tends to underestimate the risk of PF due to the growing influence of comorbidities [8]. Alternative systems, such as the PASCAL classification, which integrates anatomical risk criteria like atrial septal aneurysm (ASA) or large shunt, may better stratify older patients [9]. PFO could have clinical relevance in some older individuals due to anatomical and physiological changes. Autopsy studies reveal that PFO size and tunnel length tend to increase with age [10,11]. Additionally, elderly individuals carry a higher burden of venous thromboembolism, which may increase paradoxical embolic potential in the presence of a PFO [12,13]. Thus, while PFO occurrence is universal, its clinical significance in the elderly likely depends on features such as shunt size, ASA, and concomitant venous pathology.

Hypertension, which in our cohort had an OR of 3.88 for recurrence, is well-recognized as a stroke risk multiplier in elderly stroke populations, including those with PFO. Thus, prediction in older patients must be interpreted within a complex vascular risk landscape that is likely to have a bigger impact than the relative contribution of PFO alone.

Randomized controlled trials (RESPECT, REDUCE, DEFENSE-PFO) demonstrated significant benefit from percutaneous PFO closure in younger cryptogenic stroke patients, but generally excluded those older than 60 [14-16]. The recent age-inclusive DEFENSE-PFO subgroup analysis (mean age ~52) suggested a possible benefit in patients aged 60 and above. However, the sample size was small and not powered for age-specific outcomes [16]. Furthermore, emerging registries indicate that PFO closure in elderly patients is feasible and safe [17]. A Monzino center cohort (n = 462; 64 aged ≥65) reported no recurrent ischemic stroke at long-term follow-up, with procedural complication rates similar to those of younger patients [17].

For clinicians, these findings highlight a gray zone in management. Should older patients undergo closure, receive medical therapy alone, or be monitored with aggressive risk factor control? Current guidelines from organizations such as the AHA/ASA, AAN, SCAI, and ESO generally recommend that closure in patients over 60 be considered on a case-by-case basis [17].

Limitations

Our study has several limitations. First, its retrospective, single-center design carries inherent risks of selection bias and limits the generalizability of the findings to broader populations. Second, case identification was dependent on ICD-10 coding, which may introduce bias due to misclassification. Third, although we adjusted for major clinical confounders, unmeasured or residual confounding cannot be excluded, particularly given the observational nature of the study. Fourth, the database did not provide a detailed classification of stroke mechanism or PFO anatomical/functional characteristics (e.g., shunt size, atrial septal aneurysm, or treatment status such as PFO closure or anticoagulation), which may influence the risk of recurrent stroke. Finally, outcomes were assessed through readmission and recurrent stroke within the same health system, which may underestimate events occurring at outside institutions.

## Conclusions

In older adults with ischemic stroke, the presence of a PFO was not associated with increased risk of readmission or worse outcomes over three years. Stroke prevention strategies in this population should continue to focus on modifiable risk factors, regardless of incidental PFO findings, unless there are high anatomical risk factors that would increase risk for paradoxical embolism on a case-by-case basis.
